# Prokaryotic horizontal gene transfer within the human holobiont: ecological-evolutionary inferences, implications and possibilities

**DOI:** 10.1186/s40168-018-0551-z

**Published:** 2018-09-17

**Authors:** Ramakrishnan Sitaraman

**Affiliations:** Department of Biotechnology, TERI School of Advanced Studies, 10 Institutional Area, Vasant Kunj, New Delhi, 110070 India

**Keywords:** Microbiome, HGT, Lateral gene transfer, DNA transfer, Symbiont, Microbial ecology, Co-evolution, Natural selection, Host-microbe interaction, *Helicobacter pylori*

## Abstract

The ubiquity of horizontal gene transfer in the living world, especially among prokaryotes, raises interesting and important scientific questions regarding its effects on the human holobiont i.e., the human and its resident bacterial communities considered together as a unit of selection. Specifically, it would be interesting to determine how particular gene transfer events have influenced holobiont phenotypes in particular ecological niches and, conversely, how specific holobiont phenotypes have influenced gene transfer events. In this synthetic review, we list some notable and recent discoveries of horizontal gene transfer among the prokaryotic component of the human microbiota, and analyze their potential impact on the holobiont from an ecological-evolutionary viewpoint. Finally, the human-*Helicobacter pylori* association is presented as an illustration of these considerations, followed by a delineation of unresolved questions and avenues for future research.

"*Noah and his family were saved -- if that could be called an advantage. I throw in the ‘if’ for the reason that there has never been an intelligent person of the age of sixty who would consent to live his life over again. His or anyone else’s. The Family were saved, yes, but they were not comfortable, for they were full of microbes. Full to the eyebrows; fat with them, obese with them, distended like balloons. It was a disagreeable condition, but it could not be helped, because enough microbes had to be saved to supply the future races of men with desolating diseases, and there were but eight persons on board to serve as hotels for them. The microbes were by far the most important part of the Ark’s cargo, and the part the Creator was most anxious about and most infatuated with. They had to have good nourishment and pleasant accommodations. There were typhoid germs, and cholera germs, and hydrophobia germs, and lockjaw germs, and consumption germs, and black-plague germs, and some hundreds of other aristocrats, specially precious creations, golden bearers of God’s love to man, blessed gifts of the infatuated Father to his children -- all of which had to be sumptuously housed and richly entertained; these were located in the choicest places the interiors of the Family could furnish: in the lungs, in the heart, in the brain, in the kidneys, in the blood, in the guts. In the guts particularly. The great intestine was the favorite resort. There they gathered, by countless billions, and worked, and fed, and squirmed, and sang hymns of praise and thanksgiving; and at night when it was quiet you could hear the soft murmur of it. The large intestine was in effect their heaven. They stuffed it solid; they made it as rigid as a coil of gaspipe. They took pride in this. Their principal hymn made gratified reference to it:*

*Constipation, O Constipation,*

*The Joyful sound proclaim*

*Till man's remotest entrail*

*Shall praise its Maker’s name."*

*–* Mark Twain*, Letters from the Earth* (1909)

## Background

The human (or other multicellular host) with its symbiotic microbiota is termed the ‘holobiont’—a term coined by Lynn Margulis [1]. The tenability the holobiont view in the specific sense of its being a unit of selection was first elaborated by Zilber-Rosenberg and Rosenberg [2]. Advocates of this view point to the importance and indispensability of the human-microbial symbiosis in multiple contexts—anatomical, genetic, physiological, metabolic, developmental and immunological [3]. Critics of this view suggest that the majority of human-microbial associations that develop after birth do not fulfill the requisite criteria of vertical transmission and partner fidelity [4, 5]. It was previously suggested that this process of microbial colonization commences via the placenta in utero itself [6], but subsequent studies attributed this finding to contamination [7]. Thus, colonization by maternal microbes commences during the passage through the birth canal and later through breast milk [8–13]. Human microbial communities undergo post-natal remodeling and begin converging to the characteristic ‘adult’ profile as early as age one [14, 15]. Had this association been entirely facultative, and both microbiota and host (especially the host) capable of elaborating ‘normal’ phenotypes with little or no impact on the overall fitness, there would be no conceptual or methodological advance in using the word ‘holobiont.’ However, rapidly accumulating data in the field highlight the obligate nature of this association for humans (and other multicellular organisms) in ensuring homeostasis over the holobiont’s lifetime (surveyed in [3]). For example, it has been observed that germ-free mice, though viable, exhibit various developmental and immunological abnormalities [16–19]. We therefore suggest that one need not necessarily privilege the holistic view over a more reductionist view of the holobiont as a collection of relatively autonomous interacting modules, especially because organisms and communities are indeed constructed on a modular plan [20]. Rather, the holobiontic view is a reminder of a higher level of complexity that we cannot afford to ignore if we are to arrive at a more complete understanding of the working of multi-organismal assemblages, including ourselves.

**Fig. 1:**
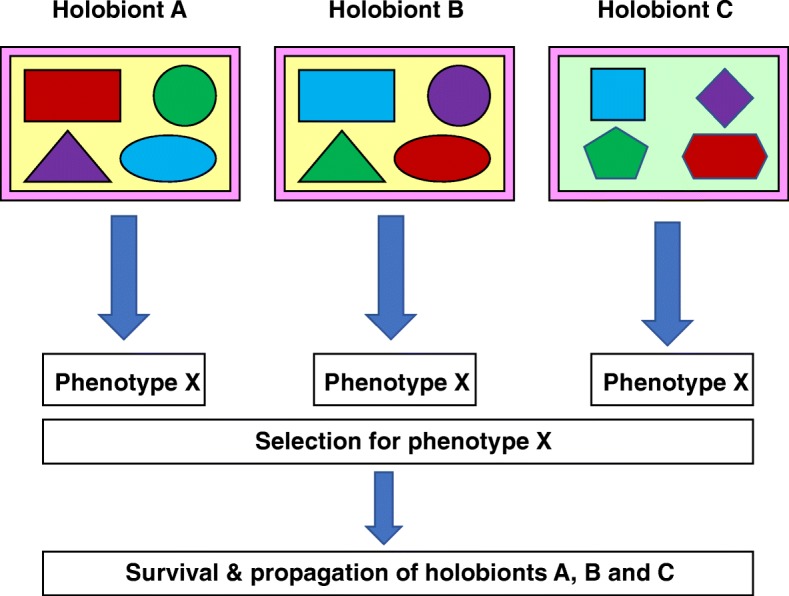
Holobiont phenotypes and selection. Holobionts A, B and C elaborate the same, positively selected phenotype ‘X’. Each multicellular host denoted by the outermost rectangle harbors four types of microbiota members (smaller shapes), with colour indicating a specific function. Hosts A and B have identical genetic backgrounds (indicated by the yellow fill color), but there is a redistribution of functions within the same components, symbolized by identical shapes but with changed colors within the outer rectangle. Host C differs from both A and B in having an entirely different genetic background (green fill color) as well as components (different shapes) but crucially retains all the functions required to produce favorable phenotype (colors are ‘conserved’). Thus, even though a holobiont phenotype (identical in all three cases) may be positively selected, the functional elements that produce this ‘favorable’ phenotype need not be identical. The findings of Lozupone et al. [24] support such phenotypic convergence under selection

In the evolutionary context, natural selection acts directly on phenotypes and only indirectly on genotypes [21]. Selection is blind to the underlying causes of a phenotype: It is merely sufficient to produce an advantageous phenotype in order to reap the benefits of increased fitness [22]. For example, the regulatory networks underlying the control of mating type in phylogenetically close species of yeast may diverge significantly in terms of how individual genes are regulated but without affecting the final output of the network [23]. Likewise, functional convergence for carbohydrate catabolism observed in the human gut microbiota has been attributed to the cooperation of different microbial species in different individuals [24] (see Fig. 1 and the section ‘HGT driven by human diet: examples of environmental selection’ below). Furthermore, the possibility of neutral or nearly neutral evolutionary changes implies that the existence of a particular phenotype may not necessarily indicate its utility in terms of prior episodes of selection or enable us to infer the nature of the selection that brought it about in every instance [25, 26]. As Syndey Brenner put it, ‘biology, because of evolution, is only the art of the satisfactory’ [20]. All that we can say with any certainty is that the evolution of multicellularity among eukaryotes (with or without prokaryotic intervention) opened up new ecological niches for other organisms, especially prokaryotes, by serving as a concentrated source of nutrients and a fairly stable habitat. Current interactions between these two groups—whether as commensals or mutualists or parasites or even facultative opportunists switching between commensalism and parasitism—offer few clues as to how these various relationships evolved and stabilized in the first instance. The acquisition, modulation and maintenance of a characteristic microbiota by multicellular hosts is probably evolutionarily ancient and conserved across diverse lineages. Characteristic and conserved microbiota are present even among representatives of basal metazoan lineages such as sponges (Phylum Porifera) [27–30] and *Hydra vulgaris* (Phylum Coelenterata) [31]. The fluctuations in microbial community composition in the initial stages of colonization in *H. vulgaris* involve host modulation by anti-microbial peptides (AMPs) resulting in the eventual stabilization of the assembled microbial communities over the lifetime of the host [32]. Recent research based on analysis of the more rapidly evolving *gyrB* gene, rather than the more slowly evolving 16S rRNA gene, has uncovered evidence of co-speciation of gut microbiota within hominid lineages—humans, chimpanzees, gorillas and bonobos. Specific clades of Bacteroidaceae and Bifidobacteriaceae identified by *gyrB* sequences have been maintained within these four hominid lineages over the order of ~ 10^5^ generations [33]. However, the distribution of Lachnospiraceae indicated that lateral microbial transfers between hominid species could also have occurred, leading to the conclusion that the human microbiota consists of both co-speciating and independently evolving microbial components.

In trying to understand how these assemblages developed, diversified and persisted over evolutionary time, we face a problem that Darwin faced in his own time: The paucity, or perhaps our own lack of recognition, of ‘transitional forms’ that could provide us a window into evolutionary innovations and ecological conditions that resulted in the relatively stable holobionts of today. In this context, we highlight the unprecedented observation that a sulfonolipid termed ‘rosette-inducing factor’ (RIF-1) produced by the aquatic bacterium *Algoriphagus machipongonensis* can induce the formation of a ‘multicellular’ rosette, an aggregate of individual cells, in the free-living, unicellular choanoflagellate *Salpingoeca rosetta* [34]. This observation raises the interesting possibility that the transition to multicellularity and the later acquisition and maintenance of a microbiome was perhaps a saltationary, rather than gradual, episode in the evolutionary history of holobionts [35]. Given that the human microbiota consists of many species of microbes whose collective numbers are of the same order as the number of cells in the human body [36, 37], it is reasonable to consider the microbiota as a reservoir of phenotypic (i.e. metabolic and physiological) diversity. Both phenotypic diversity and population size are grist to the mill of evolution and ecology. The large population size and short generation time of microbes relative to their multicellular hosts enables the rapid emergence and establishment of novel biological capabilities within a short period of time, given specific selection pressures and/or ecological opportunities.

Horizontal gene transfer (HGT; also termed lateral gene transfer [LGT] or horizontal DNA transfer [HDT]) among prokaryotes accelerates phenotypic diversification by enabling the exchange and acquisition of genetic material (and potentially, novel phenotypes), thereby bypassing the need for repeated evolution and retention of genes through vertical (lineal) transmission [38, 39]. Investigations of several prokaryotes have progressively added to our knowledge of the mechanisms of DNA transfer and uptake by conjugation, transformation and transduction. Additionally, novel modes of HGT such as membrane vesicles [40–44], nanotubes [45] and virus-like gene transfer agents (GTAs) [46, 47] have been discovered. (For recent, comprehensive overviews on HGT in prokaryotes, see references [48–50].) High rates of HGT among prokaryotes have been remarked upon. For example, Vos et al. (2015) estimated that in the case of two phylogenetically distant strains of the opportunistic plant pathogen *Pseudomonas syringae*, gene gain by HGT had occurred at a rate equivalent to 20% of the rate of point mutation [51]. Studies of the human microbiota, a large fraction of which are related to the intestinal/gut microbiota, indicate that HGT is widespread within the prokaryotic component [52–56]. HGT is probably a major contributor to protein diversification compared to gene duplication at least in some lineages of human-associated bacteria [57]. At the collective level, Liu et al. [53] identified extensive HGT averaging 43.9 HGT events per microbe among 308 members of the human microbiota for which genome sequences were available. Notably, they detected HGT between organisms located at different body sites, and gut microbiota were found to have the largest number of HGT candidates. Tamames and Moya [58] analyzed prokaryotic HGT from the available metagenomes of four different samples—a whale carcass, Sargasso sea water, farm soil and human feces. Within the last sample, the application of phylogenetic methods indicated that 1% of all open reading frames (ORFs) had been subject to HGT. Analysis of the same data by compositional methods yielded a higher estimate of 2.5–6.5% for HGT.

In this synthetic review, we recount some notable discoveries and analyses of HGT within the prokaryotic component of the human microbiota that are potentially relevant to the holobiontic context. Though both prokaryotes and eukaryotes constitute the human microbiota, this article focuses on the prokaryotic component that is far better studied, significantly more numerous [59] and possesses several well-characterized modes of HGT. Moreover, we have purposely highlighted only those instances of prokaryotic HGT that are modulated by or can potentially modulate the host phenotype, thereby necessitating a holobiontic perspective. Unstated implications of previous studies of HGT in prokaryotic pathogens vis-à-vis the microbiota are also discussed. Finally, some aspects of the association of the gastric commensal/pathogen *Helicobacter pylori* with humans are re-evaluated within the holobiontic framework. However, we do not discuss general aspects of HGT within the microbiota, HGT between the host and the microbiota or between eukaryotic and prokaryotic microbiota within the human host [60]). Finally, the dissemination of antimicrobial resistance via HGT has not been covered in this article, except in a tangential way, owing to the availability of several reviews devoted to the subject [61–64]. The terminology used in this review adheres to the conventions proposed by Marchesi and Ravel for microbiome research [65].

## Modulation of prokaryotic HGT: interplay of host and microbiota

**Fig. 2:**
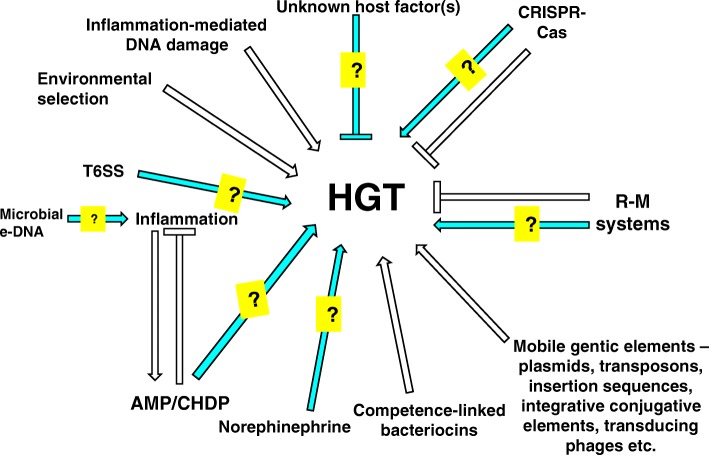
Factors influencing prokaryotic HGT within the human holobiont. A factor may enhance (—>) or inhibit (—|) HGT among the human-associated microbiota. Some factors like R-M systems may have a dual role depending on the specific context. Blue arrows with a question mark (‘?’) indicate instances wherein an effect on HGT in vivo is postulated but experimentally unverified

One line of future inquiry that would add weight to the holobiont view would be to determine how the host or the microbiota (or particular members thereof) influence the rate (tempo^1^) of prokaryotic HGT resulting in the elaboration of distinct phenotypes by the holobiont. In this section, we outline some findings that could have a bearing on our understanding of this issue. We also survey studies conducted in other contexts that reveal previously unsuspected indications of the modulation of prokaryotic HGT by the host and microbial components of the holobiont, enabling us to make some testable predictions. The information and inferences presented in this section are summarized in Fig. 2, wherein facts, theoretical possibilities and predictions are clearly demarcated.

### Putative host effectors influencing microbial HGT

Evidence for specific host effectors influencing microbial HGT is so far scanty, but there are tantalizing glimpses of possible effectors and mechanisms. A cell culture-based study of conjugation efficiency between two clinical isolates of *E. coli* (Ec77 and Ec56) found that conjugation efficiency (8.46 × 10^−5^ transconjugants/donor) decreased by a little less than twofold (4.51 × 10^−5^transconjugants/donor) when the *E. coli* strains were co-cultured with a human colorectal adenocarcinoma cell line (Caco-2), as compared to controls [66]. More intriguingly, cell-free culture filtrates of Caco-2 cells from the apical side (but not the basolateral side) were found to exert a similar suppressive effect that was ameliorated upon protease pre-treatment of culture filtrates. Therefore, the authors of that study inferred the existence of a protein (or proteins) secreted by Caco-2 cells that are capable of modulating conjugation between enteric bacteria. Whether such a factor(s) is (are) secreted in vivo within the human gut is presently unknown.

The resident microbiota are under constant selection by host innate immune system that produces cationic anti-microbial peptides (CAMP) or cationic host defense peptides (CHDP; e.g. defensins) that are capable of destabilizing bacterial membranes [67–71]. We speculate that such membrane destabilization may incidentally contribute to HGT in those microbes that are not exposed to lethal concentrations or are exposed for brief periods of time insufficient to cause lethality as they transit through different niches (e.g. through the intestinal lumen). This mode of HGT could be especially significant for species that are not naturally transformable. Also, the lysis of target organisms inevitably results in the release of DNA into the environment that is potentially available for uptake by transformation by other organisms. It is therefore possible that CHDPs are hitherto overlooked host factors that promote gene transfer by the destabilization of bacterial membranes. However, this view needs to be balanced with the finding of Cullen et al. that resident bacteria such as Bacteroidetes are relatively resistant to host CHDP action compared to transient pathogens, enabling them to survive increased CHDP secretion during a host inflammatory response to infection [72]. If resistance to CHDPs were a feature of all members of the resident microbiota, we may surmise that resident microbiota predominantly serve as recipients, rather than as donors of genetic material. Incidentally, the foregoing analysis also implies that bacterially-derived membrane-targeting antimicrobial peptides like polymyxin B, whose mode of action is similar to that of CHDPs and is being reconsidered for usage against multidrug-resistant gram-negative pathogens [73], could likewise potentiate HGT among the microbiota. Indeed, Cullen et al. also found that human-derived strains of Bacteroidetes, Firmicutes and Actinobacteria displayed significant polymyxin resistance, leading them to suggest that resident bacterial strains from the three phyla are probably resistant to host CHDPs [72]. One in vitro study to date reported that polymyxin B nonapeptide, a less nephrotoxic derivative of polymyxin B, was able to promote low levels of *E. coli* HB101 transformation (a frequency of 1 × 10^−7^/cfu) with plasmid pBR322 DNA in the absence of calcium chloride [74]. However, its effect on in vivo HGT among bacterial microbiota has not been investigated yet. Introducing marked strains into pathogen-free mice and monitoring marker exchange with and without polymyxin B administration at physiologically tolerable concentrations would perhaps address this question. Therefore, the usage of such membrane-targeting antimicrobials may have to take into account their potential to promote HGT among resident microbial families and the recent history of the patient’s exposure to antibiotics.

### Host inflammation and microbial HGT: Correlation to connection

Some data in the literature suggest that inflammation of host tissues could influence prokaryotic HGT. A study by Stecher et al. highlights a connection between inflammation and HGT among Enterobacteriaceae in the mammalian (murine) gut [75]. The authors observed that when enteric inflammation is induced in mice by streptomycin administration and followed by infection with *Salmonella enterica* serovar Typhimurium (STm), it resulted in an increase in the abundance of resident *E. coli* (mostly phylogenetic group ECOR B2) amounting to > 80% of the total intestinal bacteria. This promoted conjugal transfer of plasmid 2 (p2) from STm to *E. coli* at very high efficiencies. Thus, the apparent influence of inflammation on HGT may be coincidental, in that inflammation-associated dysbiosis in the microbiota could fortuitously lead to greater opportunities for HGT for a subset of microbes that proliferate in large numbers.

It is interesting to note that the opportunistic pathogen *Pseudomonas aeruginosa*, usually a harmless commensal of humans, produces a matrix containing extracellular DNA (eDNA) during growth in biofilms. eDNA has been found to induce human neutrophils in vitro to produce pro-inflammatory cytokines (IL-8 and IL-1β) [76]. A study in mice by Trompette et al. found that an increase in fermentable dietary fiber increased the numbers of Bacteroidetes relative to Firmicutes, with a concomitant increase in circulating short-chain fatty acids that can have an anti-allergic inflammatory effect on sites far away from the gastrointestinal tract, such as the lung [77]. These findings suggest that those members of the microbiota that are capable of modulating inflammatory states in the human host could have an effect on HGT within the microbiota. Stress hormones such as catecholamines (CAs) that are known to be involved in inflammatory responses could be a group of likely mediators, given that several bacterial species, both pathogenic and commensal, respond to CAs by changing growth rates and virulence factor expression (recently reviewed, see [78]). So far, only one report by Peterson et al. has explored the possibility of a direct link between CAs and HGT in bacteria [79]. Working with a clinical strain of *Salmonella enterica* serovar Typhimurium and *E. coli*, Peterson et al. demonstrated a threefold increased efficiency of transfer (~ 1.5 × 10^−6^ versus ~ 5 × 10^−7^ transconjugants/donor) of a conjugative plasmid from the former to the latter in vitro at physiologically relevant concentrations of norepinephrine (5 μM).

Another mechanism potentially coupling host inflammation to HGT among the microbiota could be via the production of CHDPs as a result of infection-induced inflammation (recently reviewed, see [80]). CHDPs could potentially contribute to HGT among the microbiota as discussed in the previous section, notably with less selectivity than mechanisms such as bacterial conjugation or viral transduction. However, it must be noted that CHDPs such as human LL-37 are also involved in *downregulating* the inflammatory response [80], while their overall effect on bacterial membrane permeability would be expected to remain unchanged. Thus, our postulated facilitation of gene transfer by CHDPs may not be entirely dependent on the induction of inflammation.

Recent work on *Salmonella enterica* serovar Typhimurium-induced inflammation indicates that inflammation caused by pathogens can promote HGT among different *Salmonella* strains via activation of prophages. Prophage activation results from the triggering of the bacterial SOS response in response to the DNA damage caused by free radicals released by host immune cells (e.g. neutrophils) during inflammation [81]. As free radicals would not discriminate between pathogens and commensals, the consequences for HGT are intriguing. Additionally, the effect of the bacterial SOS response on competence also bears discussion. The human pathogen and naturally competent bacterium *Streptococcus pneumoniae* lacks the LexA repressor and the SOS response. Instead, its response to DNA-damaging agents such as mitomycin C and fluoroquinolones includes the induction of competence [82]. It is tempting to speculate that microbial DNA damage due to the immune response may be involved in the development of competence in not only *S. pneumoniae* but also among members of the resident microbiota. However, it is salutary to note that the relation between HGT and the SOS response/DNA damage is also species-dependent. For example, in *Streptococcus thermophilus*, a lactic acid bacterium used in the dairy industry, the SOS response antagonizes rather than promotes the development of competence [83].

Thus, the inflammatory state that influences HGT between members of the microbiota may be a consequence of pro-inflammatory states induced in the host by a component(s) of the microbiota itself. Stated differently, the induction (or suppression) of the host inflammatory response potentially couples microbial HGT to interactions between the constituents of the holobiont. This leads to an interesting question of what comes first: Does inflammation lead to dysbiosis or does dysbiosis promote inflammation? More importantly, how resilient is the holobiont to such states, and how and when is the ‘tipping point’ reached? Can perturbations arise due to random drift, as well as during long-term environmental and physiological alterations e.g. change in diet or exercise respectively? In this context, we point out the ‘keystone pathogen hypothesis’ that suggests that certain pathogens, themselves not very numerous, could exert a disproportionate influence on both dysbiosis and inflammation [84]. The term ‘keystone’ is derived from ecology and qualifies a species that exerts a greater influence on its ecosystem than would be expected from its population size alone. In our context, we wonder if *P. aeruginosa* (discussed above) could play the role of a keystone pathogen in terms of inducing inflammation that might, in turn, modulate HGT within the microbial community.

### HGT driven by human diet: examples of environmental selection

Understandably, the human gut microbiota is subjected to environmental selection based on food sources, especially in omnivorous humans. HGT events under environmental selection can result in the preservation of gene sequences from transiting species, especially if there is a strong and persistent selection pressure (such as a reliable natural resource). This scenario was spectacularly borne out by the analysis of carbohydrate-active enzyme (CAZyme) genes in the gut microbiota of Japanese individuals. CAZyme genes encoding enzymes specific for marine algal carbohydrates (porphyranases and agarases) had been transferred from a seaweed-dwelling saprotroph (closely related to *Zobellia galactanivorans*) to *Bacteroides plebeius* within the Japanese gut microbiota [85]. These genes were not encountered in the microbial metagenomes of North American individuals (based on data available in 2010), indicating that the widespread consumption of seaweed over generations in Japan exerted selective pressure, fixing this trait enabling *B. plebeius* to exploit a reliable resource. A subsequent study indicated that HGT via an integrative and conjugative element (ICE) resulted in the horizontal acquisition by the gut bacteria *B. thetaiotaomicron* and *B. uniformis* of a polysaccharide utilization locus (PUL) that enabled these bacteria to utilize agar and carrageenan (derived from marine algae) [86]. Interestingly, these genes are present not only in Japanese individuals but also in Spanish and American individuals, indicative of microbial adaptation to the seaweed derivatives in the modern diet, especially processed foods. A different study of carbohydrate-active enzymes in the human microbiota by Lozupone et al. indicated a convergence, in terms of overall catabolic ability, i.e. phenotype but not in terms of actual species similarity or identity [24]. Interestingly, this study indicated that such functional convergence was most likely attained via HGT rather than vertical transmission, in both bacteria and archaea of the gut. It is notable that other studies have revealed extensive HGT within human gut-dwelling Bacteroidales [87, 88]. It seems that the adaptation of this important member of the gut microbiota to its ecological niche and its carbohydrate-utilizing functions are significantly predicated on prior episodes of HGT.

In 2016, Song et al. characterized a β-agarase gene (*aga1*) in the soil bacterium *Paenibacillus* sp. SSG-1 that was found to be highly similar to genes found in human oral and gut bacteria—*Paenibacillus* sp. D14 and *Clostridium* sp. D5 respectively [89]. Surprisingly, no homologs for *aga1* were found in other members of the two genera. The closest match was with the marine bacterium *Rhodopirellula sallentina* SM41, indicating HGT from a marine bacterium to the human microbiota due to seaweed consumption. As *Paenibacillus* sp. SSG-1 was isolated from soil at a site distant from a marine environment, Song et al. surmised that this was likely due to spitting or the usage of human waste as fertilizer.

We therefore suggest that HGT can serve as an ‘archiving’ mechanism establishing a reservoir of genes derived from transitory microorganisms. Perhaps this is advantageous for the holobiont as it promotes the acquisition and stabilization of useful functions within a complex microbial community with a high turnover rate. While the identity of the individuals performing the function may change over time, the function itself (and therefore, the phenotype) is retained without disruption (Fig. 1). The presence of such a ‘reservoir of functions’ may also enable adaption to altered food sources, as encountered during long migrations. In the modern context, the presence of such a reservoir may enable the transfer of antimicrobial resistance determinants to incoming pathogens that could, in the extreme, lead to the death of the holobiont and the potential dispersal or loss of these determinants. Also, note that the fitness cost of maintaining antibiotic resistance in the absence of antibiotic selection may not be too great. Antimicrobial resistance determinants are known to persist in the microbiota for significant periods of time (at least 2 years) after cessation of antibiotic administration [64, 90, 91]. On the other hand, it is also possible that these determinants contribute to fitness regardless of external antibiotic administration because they confer resistance to chemically related antimicrobial compounds produced by the members of the microbiota themselves (also refer to the discussion on polymyxin B in the section ‘Putative host effectors influencing microbial HGT’).

### Microbial modulation of HGT

The reciprocal relationship between the host and its microbiota highlighted in the preceding section does not preclude evolutionary conflicts between and within the individual members of the microbiota. The members of the microbiota also produce membrane-destabilizing agents that could potentially promote HGT in a manner analogous to CHDPs mentioned earlier. Bacteriocin production by the members of the human microbiota has been inferred by bioinformatics analyses [92–94] and also experimentally verified [95, 96]. In the case of *Streptococcus mutans*, a member of the human dental plaque biofilm, competence development is coupled to the secretion of bacteriocins, to which it is itself immune. Under these conditions, *S. mutants* was also found to be capable of taking up plasmids released by *S. gordonii* in co-culture. [97]. Likewise, the gut-dwelling *S. salivarius* couples bacteriocin production to genetic competence [98]. *S. salivarius* genomes are found to contain multiple ICEs, including those encoding bacteriocins [99]. Thus, the bacteriocin-producer, itself immune to the effects of bacteriocin production, is poised to reap the benefits of DNA release by target organisms. In addition to maintaining community composition, bacteriocin production has potentially promoted HGT, especially when it is coupled with the development of competence. An analogous situation obtains in the case of type VI secretion systems (T6SS) that are abundantly distributed in gut Bacteroidales genomes, often by ICEs, and encode secreted bacteriolytic effectors [100]. Organisms encoding such effectors also encode immunity proteins that prevent autolysis. From the viewpoint of HGT, it would be interesting to determine if any of the T6SSs in the human microbiota are co-regulated with competence development, as has been recently observed in the human pathogen *Vibrio cholerae* [101]. All the same, our view of bacteria lysing their competitors as a means of obtaining new genetic material must be tempered with an alternative possibility that DNA can also serve as a nutrient when taken up by the cellular competence machinery, at least in γ-Proteobacteria, enabling survival during the stationary phase [102]. This is especially important because Proteobacteria, though not as abundant as Bacteroidetes and Firmicutes among the human gut microbiota, nevertheless contribute to significant functional variability [103].

Prokaryotes encode a variety of mobile genetic elements, collectively termed the mobilome, that participate in HGT. Additionally, bacteriophages (and perhaps the less-studied archaeal viruses) are also significant contributors to HGT within the prokaryotic component of the human microbiota (recently reviewed, see [104]). The mobilome has been extensively analyzed in the context of antimicrobial resistance (reviewed in [56]), but there have been few studies determining their contribution to functional aspects of the holobiont. A systematic search for plasmids in the human gut microbiota found that most genes on these plasmids (other than replication-related and unclassifiable ones) happened to encode addiction modules such as toxin-antitoxin systems [105, 106] that do not have a clear functional significance for the holobiont. An extensive comparison of the mobilomes of Fijians and North Americans indicated that they differed in the abundance of specific plant starch-degrading glycosyl hydrolases [107]. This could be reliably attributed to environmental selection due to their respective diets, with the Fijian metagenomes also exhibiting a greater abundance of plant matter relative to the North American ones, underlining the potential importance of the mobilome in holobiont adaptation to varied diets.

### Specific prokaryotic barriers to HGT

Specific prokaryotic barriers to HGT are of two major types: the CRISPR-Cas and restriction-modification (R-M) systems. The CRISPR-Cas system in bacteria and archaea is analogous to an adaptive immune system in that it can prevent future productive infections by phages that have been encountered earlier. An interesting observation by Jorth and Whiteley (2012) in the case of the human periodontal pathogen *Aggregatibacter actinomycetemcomitans* links CRISPR-Cas to bacterial transformability [108]. The bacterial population of this pathogen consists of both competent and non-competent strains that breed true. However, non-competence coincidentally results in the loss of significantly greater numbers of *cas* genes compared to competent strains. Accordingly, Jorth and Whiteley suggest that rapidly changing environments would favor dynamic genomes and, therefore, competent strains, whereas stable environments would favor non-competent strains. This also means that non-competent strains that lack functional *cas* genes are vulnerable to phage infections, which could again select for competent strains. While being careful to avoid teleology, we observe that one barrier to HGT (non-competence) has concomitantly compromised another barrier that could enable HGT through phage-mediated transduction. It would be of interest to determine whether similar mechanisms that affect competence in concert with enhancing or suppressing barriers to HGT are operative among the members of the resident human microbiota.

Restriction-modification (R-M) systems are ubiquitous in both Bacteria and Archaea, as borne out by the continuously expanding database of restriction enzymes [109]. The presence of an R-M system in a bacterium (or an archaeon) largely, but not completely, prevents the stable acquisition of DNA sequences containing unprotected R-M target sequences. However, this is not entirely an all-or-none situation. Foreign DNA, even if it is a suitable target for restriction endonucleases, can be occasionally modified, and therefore protected, by DNA methyltransferases associated with R-M systems of types I–III. R-M systems (especially types I–III) themselves may be considered selfish genetic elements that ensure their propagation due to post-segregational killing, i.e. death of daughter cells that do not contain the R-M systems due to dilution of the protective (methylating) activity of the DNA methyltransferase component [110]. As selfish genetic elements, their dissemination by HGT would potentially result in their new hosts acquiring resistance to phages within the environment. Indeed, R-M systems can also occur as mobile genetic elements [111], and R-M systems, especially of type II, can be transferred horizontally [110–118]. While R-M systems may be considered barriers to HGT, the production of double-stranded DNA breaks by restriction endonucleases essentially produces substrates for recombination as well [119]. Also, note that single-stranded DNA is largely immune to restriction enzyme digestion, even if is unmodified and contains target sequences recognized by the enzyme. It has been proposed that R-M systems (and by extension, similar barriers to HGT) could serve to maintain species stability [120] as well as that of clades within a single species [121]. However, this view must be counterbalanced by the observations of Oliveira et al. [122] who found that HGT is positively correlated with genome size and numbers of R-M systems encoded by the genome. R-M systems also tended to limit HGT between phylogenetically close organisms containing non-cognate R-M systems, while permitting genetic exchange between evolutionarily distant organisms encoding cognate R-M systems.

## The human-*Helicobacter pylori* association: a case study in shades of grey

Our view of host-microbe interactions has historically evolved in the background of what has been termed a ‘dualistic framework’ of ‘good’ versus ‘evil’ [123]. Briefly, these include the initial concept of pathogens versus host, subsequent ideas of ‘good’ and ‘evil’ members of the microbiota and inflammatory and non-inflammatory states in the host. However, as Eberl [123] has suggested, both microbes and their host exhibit multiple phenotypes in a variety of contexts, with the outcomes spanning a continuum, rather than two distinct, non-overlapping categories. We suggest that the case of *Helicobacter pylori*, an ancient [124] and a prominent member of the human stomach microbiota [125, 126] is highly supportive of this viewpoint. A recent analysis of historical patient data indicates that it infects more than half the human population [127]; however, the majority of *H. pylori* carriers are asymptomatic. Infected individuals present with varying degrees of gastric inflammation, and a progressively decreasing minority of hosts develop peptic/duodenal ulcers or gastric cancer or lymphoma of mucosa-associated lymphoid tissue.

The co-existence of *H. pylori* with its human host, the chronicity of its infection and the variable nature of the clinical outcomes for the host indicate that co-evolution of *H. pylori* and humans is an ongoing process and perhaps reflects a ‘transitional form’ of holobiont evolution alluded to in the introduction. It has been suggested that the view of *H. pylori* as a human pathogen is due to its discovery in a pathogenic context and that this association may be viewed as the result of a trade-off between costs and benefits for the human holobiont [128]. Indeed, there are indications that *H. pylori* has a protective effect against childhood diarrheal diseases [129]. Gastric inflammation caused by *H. pylori* infection enhances the gastric immune response against the cholera vaccine [130]. *H. pylori* infection is also positively correlated with enhanced protection against tuberculosis [131, 132]. Therefore, it is possible that the cost of developing diseases later in life due to *H. pylori* infection is offset in evolutionary terms, by the increase in the likelihood of the human host attaining reproductive age [133].

The spectrum of clinical outcomes upon *H. pylori* infection, especially the development of gastric cancer due to chronic inflammation later in life is strongly correlated with the presence of a pathogenicity island (PAI) designated *cag* (cytotoxin-associated gene) that encodes a type IV secretion system (T4SS) and has probably been acquired by some strains via HGT [134, 135]. The *cag*PAI encodes the oncogenic CagA protein that can be translocated via the T4SS into host cells with which *H. pylori* comes into close contact. This has the effect of tilting the balance in favor of increased inflammation and gastric cancer risk, but it is not known whether increased and chronic inflammation due to *H. pylori* infection influences HGT in *H. pylori* and/or other members of the human microbiota. A subset of *H. pylori* strains also encodes one or more T4SSs that can transfer DNA to other strains as well as related species like *Campylobacter jejuni* in vitro [136]. Given that the primary habitats of these two bacteria are different—*H. pylori* inhabiting the stomach and *C. jejuni* the small intestine—this raises the possibility of HGT between transiting and resident bacterial species, ensuring the dissemination of genes from one ecological niche to another. Rohrer et al. determined that the *comB* T4SS (present in all *H. pylori* strains) was necessary for plasmid uptake in recipient *H. pylori* cells by both transformation and conjugation [137]. The genomes of some *H. pylori* strains contain “plasticity zones” harboring transposons that encode, among other elements, the *tfs3* [138, 139] and *tfs4* [140] type IV secretion systems. However, the components of these two T4SSs do not seem to influence HGT [137].

While *H. pylori* is naturally competent [141, 142], it also encodes exceptional numbers of R-M enzymes—more than twenty on average across all known strains (see https://tinyurl.com/y9pntzw3). It exhibits considerable strain diversity across geographical locations [143] and even within a single human host [144]. Given the large numbers of R-M enzymes that each strain encodes, trans-species HGT would be expected to preferentially involve *H. pylori* in the role of DNA donor, rather than an acceptor, as was seen with the case of *C. jejuni* (above). Even among *H. pylori* strains, we might expect that the success of HGT might depend on the extent of genetic relatedness (i.e. sharing the same R-M complement). Incidentally, the plasmid transfer experiments of Rohrer et al. [137] involving unrelated clinical isolates of *H. pylori* suggest that the numerous R-M systems are not insuperable barriers to DNA transfer in *H. pylori*. Bubendorfer et al. (2016) carried out a detailed analysis of inter-strain transfer of genomic DNA fragments and their patterns of integration into the recipient genome via homologous recombination using carefully chosen *H. pylori* strains in an attempt to address this issue [145]. Their study, conducted entirely in vitro, indicated that recipient R-M systems do not seem to affect the integration of homologous DNA, even though they seem to be effective barriers against the integration of heterologous DNA.

## Conclusions

The role of intra-prokaryotic HGT in the overall survival and propagation of the human (and other) holobionts is barely beginning to be understood, not least because of the immense technical, logistic and ethical challenges involved. HGT could potentially ensure the wider dissemination (and preservation) of genes derived from rare or transient/extraneous members of the microbial community and restore functions that would be otherwise compromised as a result of gene/species loss. Moreover, human cultural evolution may also have already impacted HGT in unforeseen methods. The invention of sewage networks that concentrate unprecedented quantities of human and animal waste before eventual disposal (with or without treatment) into water bodies has presented microorganisms with a nutrient-rich environment on a vast scale that was absent for most of human history and may have affected microbial HGT in unknown ways [146–148]. The intensive farming of food animals (both terrestrial and aquatic) prevalent in more industrialized nations uses antibiotics on a large scale, leading to the increased abundance of antibiotic resistance determinants as well as an increased incidence of HGT [52, 149]. Notably, such activities may eventually influence populations that are located at significant distances downstream or along the same coast (in the case of marine environments) in unprecedented ways [150, 151].

The holobiontic perspective has the signal merit of bringing into sharp focus the value of functional studies of the microbiota as a necessary and informative complement to studies based on 16S rRNA gene-based diversity analysis. Functional information integrated with other available ‘omics’ technology platforms, especially proteomics and metabolomics, could be more informative in identifying interactions that cooperate to produce the holobiont phenotype. Metagenomic sequencing of microbial communities can, in principle, enable identification of HGT events within the population under scrutiny using computational methods. However, a limiting factor in detecting HGT in these communities is our ability to assign a source organism for the short sequence reads typical of metagenomic samples. Recent work assembling genomes entirely from metagenomic data demonstrates that such a reconstruction is indeed possible [152–154]. Microbial culturomics—the high-throughput culture of organisms in complex communities—can potentially provide us with reference genomes for comparison and is beginning to be applied to the human microbiota [155–157].

As noted in the first section, some estimates of the extent of HGT in individual microbes as well as microbial communities are available. However, compared to *detecting* HGT events *post facto*, estimating their *rate* presents additional problems. The mechanisms of HGT and their frequency of occurrence are dependent on the species under consideration as well the environmental conditions (biotic and abiotic) prevailing at a given time. In order to determine changes in rates above a ‘background’ as a result of a particular intervention, it would be necessary to monitor HGT between donors and recipients before and after the said intervention. Clearly, at the present time, such studies can be conducted with individual organisms (depending on our ability to culture them), but not with entire communities. Additionally, quantitative assessments made based on particular species may not be applicable to others. Selection pressures themselves vary in terms of kind, degree and duration, probably resulting in a wide variation of transfer rates throughout the holobiont’s lifetime. The influence of second-order effects i.e., changes that alter HGT rates, is also difficult to quantify across the board for a highly diverse and still insufficiently characterized, dynamic microbial community. For example, it has been discovered that *Roseburia hominis*, a firmicute symbiont of the human gut, exhibits an upregulation of the transcription of genes related to plasmid mobilization/conjugation 14 days after being administered to germ-free mice [158]. Therefore, information about a ‘background’ rate of HGT may not be as informative as we might expect. Rather, it would be more informative to determine how particular instances of HGT have contributed to overall functionality and fitness, thereby strengthening the associations that constitute the holobiont.

Microbiological research has afforded us unparalleled glimpses of the hidden lives led by DNA in prokaryotic communities comprised of billions of individuals, not only in its journey down lines of lineal descent but also across phylogenetic groups. Both microbiology and medicine have come a long way since Mark Twain wrote his corrosive satire quoted in the beginning. It is increasingly clear that humans do not merely provide ‘*sumptuous housing*’ for the microbes but also receive significant ecosystem services in return. And more importantly, ‘*germs*’ causing ‘*desolating diseases*’ are by no means representative of the salient contributions of a vast number of microbes either. However, precisely because research efforts have focused intensely on ‘germs’ for more than a century, much information is available that could be leveraged to better understand the role of HGT in maintaining holobiont homeostasis. As the case of *H. pylori* indicates, some of the implications of this information may be underappreciated due to its focus on pathogenesis. Thus, we cannot help but appreciate Mark Twain’s prescience in discerning that the microbes (harmful or not) were indeed ‘*the most important part of the Ark’s cargo*,’ and affirm that rapidly accumulating knowledge of the many functions of the microbiota in diverse multicellular organisms provides ‘*man’s remotest entrail*’ with sufficiently weighty reasons ‘*to praise its Creator’s name*.’

To reiterate, positive or negative selective pressures can impact the holobiont simultaneously and at multiple levels of complexity. The choice of the holobiont as a unit of selection does not exclude other units of selection at lower levels of complexity—human, microbial, genomic or genetic. In this article, we have purposely highlighted only those instances of HGT modulation that couple the human host and members of the microbiota, thereby supporting the current usage of the term ‘holobiont’ [159, 160]. Understandably, many questions remain unaddressed. What is the relative contribution of different modes of HGT to the overall fitness of the holobiont? Are there major and minor contributors to HGT? Does the relative proportion of contributions to HGT by multiple mechanisms vary over the lifetime of the human (or other) host and, if so, due to which factors? Does the identity of the predominant HGT mode(s) vary across ecological niches (body sites) and does environmental selection impact the preponderance of one mode over another? Are certain members of the microbiota dominant drivers of HGT and if so, under what circumstances? How do the multicellular host and the unicellular eukaryotic component of the microbiota influence and contribute to these processes within the prokaryotic component? Answers to these questions may significantly influence our future view of the human holobiont in health and disease, within families, communities and entire cities and perhaps impact future strategies for therapy, health maintenance and improvement.

## Abbreviations

AMP: Anti-microbial peptide
CA: Catecholamine
*cag*: Cytotoxin-associated gene
*cag*PAI: *cag* pathogenicity island
CAMP/CHDP: Cationic anti-microbial/host defense peptide
Cas: CRISPR associated (gene)
CAZyme: Carbohydrate-active enzyme
CRISPR: Clustered regularly interspaced palindromic repeats
eDNA: Extracellular DNA
HDT: Horizontal DNA transfer
HGT: Horizontal gene transfer
LGT: Lateral gene transfer
ORF: Open reading frame
RIF-1: Rosette inducing factor-1
R-M: Restriction-modification
T4SS: Type IV secretion system
T6SS: Type VI secretion system

## Footnotes

1 Tempo and mode are terms first introduced in 1944 by the paleontologist George Gaylord Simpson in his book *Tempo and Mode in Evolution* [161]
